# Brief report: Ketamine‐assisted “bridge therapy” for opioid tapering in complex cases

**DOI:** 10.1111/ajad.70131

**Published:** 2026-01-28

**Authors:** Mariana C. de Oliveira, Cláudia C. de Araujo Palmeira, André B. Negrão, Ziyad A. Hadi, Julia R. Arana, Rita de Cássia Ferreira Duarte, Joice L. P. da Silva, Gustavo K. Matsui, João F. D. Rapozero, Douglas H. Crispim, André Malbergier, João M. Castaldelli‐Maia

**Affiliations:** ^1^ Instituto Perdizes (IPER), Department of Psychiatry Hospital das Clínicas da Faculdade de Medicina da Universidade de Sao Paulo São Paulo 05021‐001 São Paulo Brazil; ^2^ Department of Psychiatry Medical School São Paulo University São Paulo Brazil

## Abstract

**Background:**

Opioid use disorder (OUD) presents major challenges, especially when combined with chronic pain and psychiatric comorbidities. A 25‐year‐old woman with OUD, chronic pain, and major depressive disorder underwent an 8‐week protocol of intravenous ketamine infusions (0.5 mg/kg weekly) as an adjunct to opioid tapering.

**Results:**

Methadone dosage was reduced by 50%, with improved pain and craving control, reduced withdrawal symptoms, and greater psychotherapy engagement.

**Discussion and Conclusions:**

Ketamine‐assisted “bridge therapy” treatment may support opioid tapering in complex cases.

**Scientific Significance:**

This is a novel report to document a multifaceted improvement in this specific comorbidity cluster.

## INTRODUCTION

Opioid use disorder (OUD) is a chronic, relapsing condition associated with substantial morbidity, mortality, and functional impairment.^1^ Comorbid chronic pain and psychiatric disorders, particularly major depressive disorder (MDD), are common and are associated with poorer treatment outcomes.^2^ This triad may create a self‐perpetuating cycle in which pain exacerbates depression, depression reduces pain tolerance and treatment adherence, and opioid use provides only transient relief while worsening long‐term outcomes.^3^

Medications for OUD (MOUD), including methadone, buprenorphine, and naltrexone, remain the gold‐standard treatment.^1^ However, determining the optimal timing and feasibility of opioid tapering is clinically challenging in patients with complex, treatment‐refractory presentations.^2^ In this context, adjunctive strategies that support engagement, symptom control, and readiness for tapering may be considered.

Ketamine, an N‐methyl‐d‐aspartate (NMDA) receptor antagonist, has pharmacological properties that may be relevant to this clinical overlap.^4^ Its analgesic and anti‐hyperalgesic effects, rapid antidepressant action in treatment‐resistant depression, and potential to reduce withdrawal symptoms and craving have led to interest in its use as an adjunctive intervention.^4^, ^5^, ^6^ Nevertheless, empirical evidence supporting ketamine‐facilitated opioid tapering in patients with OUD remains limited.^7^ We present a case in which a structured intravenous ketamine protocol was temporally associated with methadone dose reduction in a young woman with OUD, chronic pain, and MDD.

## CASE DESCRIPTION

A 25‐year‐old woman reported severe right‐sided pain since age 7, with monthly recurrent episodes. At 18, she maintained pain crises and developed functional pain disorder, later evolving into a clinical presentation consistent with somatic symptom disorder, leading to frequent emergency department visits where morphine was repeatedly prescribed. Family history was negative for substance use disorders, psychiatric conditions, or chronic pain syndromes. Prolonged exposure resulted in tolerance and subsequent OUD. Substitution therapy with methadone was initiated, but by age 19, she developed a pattern of methadone abuse.

She was referred for treatment at the specialized Addiction and Pain Management Service due to several unsuccessful attempts to manage OUD. At the time of admission, her primary treatment goal was to regain functionality, stating: “I want my life back”. Physical examination and vital signs revealed no significant abnormalities, and neurological examination was unremarkable. Extensive medical and neurological workup, including imaging and laboratory investigations performed over the years, did not reveal an active nociceptive or structural pathology to account for pain severity. The primary diagnostic challenge involved distinguishing pain related to an identifiable nociceptive condition (potentially warranting opioid therapy) or a neuropathic condition, from nociplastic pain, which was further complicated by a substantial emotional component.

Upon intake, the patient met DSM‐5 criteria for OUD, supported by a high score on the Alcohol, Smoking and Substance Involvement Screening Test (ASSIST) for opioid use (36), reflecting daily use, persistent craving, significant functional impairment, and repeated unsuccessful attempts to control use. She resisted proposed therapeutic interventions, including opioid tapering, and did not engage in group activities. Instead, she persistently demanded increased opioid dosages for pain control, which limited the treatment options. Her MDD was refractory to multiple antidepressant trials, including selective serotonin reuptake inhibitors, serotonin–norepinephrine reuptake inhibitors, and mirtazapine.

After 12 months of initial follow‐up, intravenous ketamine (0.5 mg/kg) was initiated based on depression protocols, administered as a 45‐min continuous infusion weekly for 8 weeks.^5^ The aim was to improve pain, MDD, and facilitate opioid tapering. Infusions were conducted in a day hospital under the supervision of an anesthesiologist, with continuous monitoring of vital signs, including pulse oximetry and noninvasive blood pressure. Transient hypertension and one episode of nausea resolved without intervention. Dissociative symptoms were mild and transient, as measured by the Clinician‐Administered Dissociative States Scale 6‐Item (CADSS‐6). Craving for opioids decreased progressively during sessions, and no ketamine craving was reported, as assessed clinically. Baseline and posttreatment labs showed no abnormalities. The patient was discharged the same day after 4 h of observation following each infusion.

Concurrent with the initiation of ketamine infusions, a gradual methadone taper was initiated after an initial 2‐week stabilization period. Thereafter, dose reductions were implemented in a stepwise manner, with decreases of approximately 25% (≈15 mg) every 2 weeks. This resulted in a reduction from 60 mg/day to 45 mg/day by week 3 and to 30 mg/day by week 5, completing a 50% reduction over an 8‐week period. Further dose reductions were intentionally deferred at that stage to preserve the stabilizing role of MOUD and to minimize the risk of withdrawal symptoms or clinical destabilization associated with more aggressive tapering. Following completion of the ketamine protocol, methadone was maintained at 30 mg/day and subsequently discontinued gradually over the following 6 months during outpatient follow‐up.

This tapering was well‐tolerated and temporally associated with improved pain control and no significant withdrawal symptoms reported as measured by the Clinical Opiate Withdrawal Scale (COWS), a reduction in opioid abuse, and enhanced engagement and response to psychotherapy. No additional medication was added during this process beyond the patient's existing regimen, which included duloxetine 120 mg/day and pregabalin 300 mg/day.

Clinical improvement was reflected across multiple measures. The Brief Pain Inventory (BPI) score decreased from 7 to 2. The Patient Health Questionnaire (PHQ‐9) score dropped from 17 to 4 after the first ketamine session and remained stable. Health‐related quality of life domains improved as assessed by the Short Form‐36 Health Survey (SF‐36), at 6‐month follow‐up (Table 1). These psychological improvements appeared to create a positive cascade effect: as her mood stabilized and emotional limitations decreased, she became more receptive to psychotherapeutic interventions and demonstrated increased motivation for opioid tapering.

**Table 1:** Short Form Survey (SF‐36) scores before and after the 8‐week ketamine treatment protocol.

SF‐36 initial	SF‐36 final
Physical functioning: 35	Physical functioning: 85
Role limitations due to physical health: 50	Role limitations due to physical health: 100
Role limitations due to emotional problems: 66.7	Role limitations due to emotional problems: 100
Vitality: 50	Vitality: 85
Mental health: 64	Mental health: 88
Social functioning: 62.5	Social functioning: 75
Bodily pain: 45	Bodily pain: 72
General health: 35	General health: 72

*Note*: The table illustrates changes in physical, emotional, and social functioning, as well as bodily pain and general health perception (0–100; higher = better).

Weekly urine drug screening revealed no illicit opioid use. The patient continued with monthly medical follow‐up and weekly psychotherapy. She maintained the reduced methadone dose and, with her continued clinical improvement, was able to successfully discontinue methadone completely over the subsequent 6 months.

## DISCUSSION

We report a case in which a structured, 8‐week intravenous ketamine protocol was temporally associated with methadone tapering in a patient with the challenging triad of OUD, chronic pain, and MDD. Concurrent improvements in pain, mood, and engagement with care were observed during the intervention period. Rather than establishing causality, this report highlights a potential supportive role for ketamine in carefully selected patients whose clinical course has remained refractory to conventional approaches.

Our observations complement the limited existing literature on ketamine use in addiction. While Krupitsky et al. demonstrated efficacy in achieving abstinence in heroin‐dependent individuals, this case illustrates a different paradigm: the use of ketamine to facilitate gradual, medically supervised tapering rather than immediate cessation.^8^ Similarly, while Jovaisa et al. showed ketamine's utility in managing acute withdrawal symptoms,^9^ this case describes clinical effects observed beyond the acute withdrawal phase.

A recent systematic review indicates that ketamine use in OUD is generally well tolerated and associated with reductions in craving, withdrawal severity, and depressive symptoms, although further large‐scale trials are needed.^7^ The weekly 0.5 mg/kg infusion schedule used here represents a pragmatic approach that balances sustained antidepressant effects—supported by meta‐analysis evidence^5^—with the outpatient feasibility needed in patients requiring concurrent chronic pain management. Whereas typical protocols for isolated depression or withdrawal management often employ more frequent initial dosing, the weekly 0.5 mg/kg schedule used in this case prioritized feasibility in an outpatient setting.^5^, ^8^

Given concerns regarding ketamine's abuse potential, careful risk–benefit assessment was essential.^10^ In this carefully selected patient—who had no history of illicit or polysubstance use and received treatment in a closely monitored hospital setting—the potential benefits were judged to outweigh the associated risks. Ongoing monitoring for craving and cardiovascular safety, integration within a multidisciplinary treatment framework, and prior psychiatric screening to exclude psychotic or manic disorders were considered essential to minimize potential harms.^4^

Within this framework, ketamine may be conceptualized as a *bridge therapy*—defined here as a time‐limited, adjunctive intervention intended to support the transition from high‐dose opioid treatment to lower doses by reducing symptom burden, and enhancing readiness for change, while MOUD continues as the maintenance, evidence‐based foundation of care. In contrast to MOUD, which are intended for long‐term use, ketamine in this context is applied transiently to facilitate engagement during a critical transitional period.

### Limitations and future research directions

This single case report has important limitations, including the inability to establish causality or generalizability. Moreover, the intensive clinical attention and supportive environment during ketamine infusions may have influenced the outcomes independently of the drug's pharmacological effects. Further studies are needed to confirm and expand these findings and to evaluate ketamine's efficacy in patients with isolated OUD, clarifying whether the benefits observed in this complex case can be generalized to broader addiction populations.

## CONCLUSION

In conclusion, this case report describes a clinical observation in which intravenous ketamine was used as a supportive, time‐limited adjunct during methadone tapering in a patient with complex, treatment‐refractory comorbidities. Although causal inferences cannot be drawn, these observations may inform future research on integrated approaches to these intersecting conditions, particularly in clinical contexts characterized by poor prognosis and limited treatment options.

## AUTHOR CONTRIBUTIONS

*Conceptualization*: Mariana Campello de Oliveira, Cláudia C. de Araujo Palmeira, and João Maurício Castaldelli‐Maia. *Methodology*: Mariana Campello de Oliveira, João Felippe Donaire Rapozero, and João Maurício Castaldelli‐Maia. *Formal analysis*: João Maurício Castaldelli‐Maia, Gustavo Kenji Matsui, André Malbergier, and Julia Rodrigues Arana. *Writing—original draft preparation*: Mariana C. de Oliveira, and Ziyad Abdel Hadi. *Writing—review and editing*: Mariana Campello de Oliveira, André Brooking Negrão, and Joice L. P. da Silva. *Visualization*: Douglas Henrique Crispim, Ritade Cássia Ferreira Duarte. *Supervision*: João Maurício Castaldelli‐Maia and André Malbergier. All authors have read and agreed to the published version of the manuscript.

## CONFLICT OF INTEREST STATEMENT

The authors declare that there are no conflict of interests.

## ETHICS STATEMENT

Institutional Review Board approval: CAAE: 85860224.2.0000.0068.

## Data Availability

Due to the nature of the research and ethical considerations, supporting data are not available.
